# Quantitative Gait Analysis Reveals Distinct Patterns Associated With Pyramidal Involvement in Amyotrophic Lateral Sclerosis: A Cross‐Sectional Study

**DOI:** 10.1002/brb3.71498

**Published:** 2026-05-19

**Authors:** Nan Hu, Ming Qi, Ning Su, Dingding Zhang, Jiangxia Zhang, Yun Xu, Kai Wang, Yuming Xu, Zhenzhong Li, Bo Hu, Lihua Wang, Bo Wu, Lan Chu, Yinzhou Wang, Hong Jiang, Zhengqi Lu, Jian Wu, Xiangmin Fan, Fei Han, Feng Tian, Jing Yuan, Mingsheng Liu, Yicheng Zhu

**Affiliations:** ^1^ Department of Neurology Peking Union Medical College Hospital Beijing China; ^2^ Medical Research Center Peking Union Medical College Hospital Beijing China; ^3^ Department of Neurology Nanjing Drum Tower Hospital Nanjing China; ^4^ Department of Neurology The First Affiliated Hospital of Anhui Medical University Hefei China; ^5^ Department of Neurology The First Affiliated Hospital of Zhengzhou University Zhengzhou China; ^6^ Department of Neurology The Second Hospital of Hebei Medical University Shijiazhuang China; ^7^ Department of Neurology, Union Hospital, Tongji Medical College Huazhong University of Science and Technology Wuhan China; ^8^ Department of Neurology the Second Affiliated Hospital of Harbin Medical University Harbin China; ^9^ Department of Neurology West China Hospital of Sichuan University Chengdu China; ^10^ Department of Neurology The Affiliated Hospital of Guizhou Medical University Guiyang China; ^11^ Department of Neurology Fujian Province Hospital Fuzhou China; ^12^ Department of Neurology Xiangya Hospital of Central South University Changsha China; ^13^ Department of Neurology the Third Affiliated Hospital of Sun Yat‐Sen University Guangzhou China; ^14^ Department of Neurology Beijing Tsinghua Changgung Hospital affiliated to Tsinghua University Beijing China; ^15^ Institute of Software Chinese Academy of Sciences Beijing China; ^16^ The CARE Technology Co., Ltd Beijing China

**Keywords:** amyotrophic lateral sclerosis, gait, UMN dysfunction

## Abstract

**Objective:**

To dissect specific gait abnormalities associated with upper motor neuron (UMN) dysfunction in amyotrophic lateral sclerosis (ALS) by controlling for overall disease severity and to develop a multivariate classification model.

**Methods:**

We performed 3D gait analysis on 118 ALS patients and 1796 healthy controls (HC). ALS patients were categorized into those with ALS with UMN dysfunction((ALS‐UMN), *n* = 70) and those without ALS without UMN signs ((ALS‐Numn), *n* = 48) lower limb UMN signs based on neurological examination. Gait parameters were compared, and their association with UMN involvement was analyzed using partial correlation (controlling for ALSFRS‐R score) and machine learning models (Random Forest and Least Absolute Shrinkage and Selection Operator (Lasso) regression).

**Results:**

Compared with HC, ALS patients exhibited widespread gait deterioration (e.g., reduced speed, increased step width, *p* < 0.001). After controlling for ALSFRS‐R, specific parameters, including reduced stride, increased step width, prolonged double support, and elevated gait cycle time asymmetry, remained independently associated with UMN severity (PENN score, *p* < 0.01). A multivariate model incorporating key features demonstrated fair discriminative ability for identifying ALS‐UMN patients, with an area under the curve (AUC) of 0.690, a sensitivity of 0.816, and a specificity of 0.418.

**Conclusion:**

Quantitative gait analysis reveals a distinct spatiotemporal pattern linked to UMN dysfunction in ALS. A model based on gait features shows potential, particularly high sensitivity, for identifying patients with pyramidal signs, supporting the exploratory utility of objective gait metrics for motor phenotyping in ALS, pending external validation.

## Graphical abstract

This cross‐sectional study used 3D quantitative gait analysis in 118 ALS patients and 1796 healthy controls. After controlling for disease severity (ALSFRS‐R), specific gait parameters—including reduced stride length, increased step width, prolonged double support, and elevated gait cycle time asymmetry—were independently associated with lower limb upper motor neuron (UMN) burden (PENN score). A multivariate model incorporating stride length and ALSFRS‐R showed fair discriminative ability for identifying patients with pyramidal signs (AUC = 0.690, sensitivity 0.816). These findings demonstrate that objective gait metrics can capture UMN‐specific locomotor features in ALS, supporting their exploratory utility for motor phenotyping and screening.

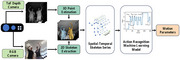

**Abbreviations**
AI: Artificial intelligence
ALS: Amyotrophic lateral sclerosis
ALSFRS‐R: The revised ALS functional research scale
AUC: Area under curve
BMI: Body mass index
EMG: Electromyography
HC: Healthy controls
IQR: Interquartile range
LMN: Lower motor neuron
MND: Motor neuron disease
MRC: The Medical Research Council
PUMCH: The Peking Union Medical College Hospital
ROC: Receiver operating characteristic
SD: Standard deviation
UMN: Upper motor neuron

## 1 Introduction

Movement analysis has evolved into a vital tool not only in sports science but also in clinical neurology for diagnosing and monitoring disorders of motor control. Gait, a complex motor indicator, requires the integrated function of upper and lower motor neurons (UMN and LMN), as well as limb muscle strength. Gait disturbances significantly impair quality of life and are associated with an elevated risk of falls, mortality (Veronese et al. 2018; Studenski et al. 2011; Rodríguez‐Molinero et al. 2019) and dementia (Taniguchi et al. 2017; Beauchet et al. 2016; Verghese et al. 2002).

Amyotrophic lateral sclerosis (ALS), a prototypical motor neuron disease (MND) (Kiernan et al. 2011), is characterized by progressive degeneration of both UMN and LMN (Grad et al. 2017). These dual involvement leads to a heterogeneous clinical presentation, including variable patterns of weakness, spasticity, and gait impairment (Tahedl et al. 2024; Abidi et al. 2021; Radovanović et al. 2014). The specific contributions of UMN dysfunction (e.g., spasticity and impaired coordination) versus LMN dysfunction (e.g., weakness, atrophy) to gait disturbance in ALS remain poorly qualified. Current clinical assessments, such as the revised ALS functional rating scale (ALSFRS‐R) (Cedarbaum et al. 1999), provide global functional scores but lack the granularity to disentangle these distinct neural substrates.

Quantitative gait analysis studies offer a promising avenue to address this gap. While previous studies have confirmed that ALS patients exhibit altered gait parameters correlating with overall disease severity (Sukockienė et al. 2021; Iancu Ferfoglia et al. 2016), there is a paucity of research systematically linking specific gait abnormalities to the degree of UMN or LMN involvement in the limbs. Advanced motion capture systems, like the 3D Azure Kinect camera coupled with machine learning algorithms, enable the extraction of detailed, multi‐parametric gait signatures that may capture subtle, clinically relevant features beyond the resolution of standard observation.

Therefore, in this cross‐sectional study, we aim to: (1) comprehensively characterize the gait abnormalities in ALS patients compared to healthy controls (HC) using a 3D vision‐based system; and (2) investigate whether specific quantitative gait parameters are associated with clinical markers of disease severity, and more importantly, with signs indicative of UMN involvement in the lower limbs. Our findings may facilitate the development of objective, technology‐aided biomarkers for stratifying ALS patients (Morioka et al. 2022; Morioka et al. 2022), and monitoring disease progression.

## 2 Methods

### 2.1 Participants

#### 2.1.1 Patients With ALS

Patients diagnosed as definite or probable ALS according to the revised El Escorial criteria (Brooks et al. 2000) were consecutively recruited between March, 2024 and November, 2024 in the Department of Neurology, Peking Union Medical College Hospital (PUMCH). All patients underwent a comprehensive clinical assessment, including demographic data collection, detailed neurological examination, and needle electromyography (EMG). Disease severity was quantitative using the ALSFRS‐R score (Cedarbaum et al. 1999). Limb muscle strength was evaluated bilaterally using the medical research council (MRC) score, including assessment of the following muscle actions: neck flexion and extension, bilateral shoulder abduction, elbow flexion, elbow extension, wrist flexion, wrist extension, thumb abduction, hip flexion, knee flexion, knee extension, ankle dorsal extension, and ankle plantar flexion. The total MRC score was 120.

Exclusion criteria were: (1) inability to complete the gait tests even with the use of a walking aid (e.g., cane, walker); (2) predominant respiratory dysfunction (forced vital capacity, FVC < 50% predicted) that could limit physical activity; (3) comorbid central nervous system, orthopedic, or other medical conditions known to significantly affect gait; and (4) cognitive impairment preventing understanding of test instructions. The use of any walking aid during testing was recorded. Informed consent was obtained from all participants and/or their legal guardians.

#### 2.1.2 HC

Data from age‐ and sex‐matched HC were obtained from an unpublished nationwide, multi‐center database established between September, 2021 and March, 2023. Control participants had no self‐reported neurological, musculoskeletal, or other conditions affecting gait. To address potential confounding due to differences in recruitment timeline and geographic distribution between groups, we performed propensity score matching based on age, sex, and body mass index (BMI) for all primary case‐control comparisons, The study was approved by the Institutional Review Board of PUMCH.

#### 2.1.3 Clinical Subgroup Definition

To investigate the association between gait patterns and UMN dysfunction, ALS patients were categorized into two subgroups based on the neurological examination of the lower limbs: (1) ALS with UMN signs (ALS‐UMN): presence of increased muscle tone (modified Ashworth scale ≥ 1) and/or elevated tendon reflex and/or positive pathological reflexes (e.g., Babinski sign) in at least one lower limb; and (2) ALS without UMN signs (ALS‐nUMN): absence of the above signs.

Besides, the burden of UMN involvement of the lower limbs was semi‐quantitatively assessed according to the Penn UMN score (PENN score) (Woo et al. 2014): Increased knee jerk reflex was scored as 1 point; increased ankle jerk reflex was scored as 1 point; pathological signs: bilateral positivity was scored as 2 points, unilateral positivity as 1 point, and negativity as 0 points; increased muscle tone (modified Ashworth scale ≥ 1): bilateral increase was scored as 2 points, unilateral increase as 1 point, and no increase as 0 points. The total score range was 0–6 points.

### 2.2 Gait Acquisition and Processing

Gait was assessed using the Ready‐GO system (Beijing CAS‐Ruiyi Information Technology Co., Ltd.), a medical device certified by the National Medical Products Administration of China. The system employs a Microsoft Azure Kinect depth camera (30 Hz sampling frequency) positioned frontally to capture whole‐body movement. The system's proprietary software uses a combination of RGB and depth data to reconstruct a 3D skeletal model (25 joints) in real‐time. A deep learning‐based pipeline (utilizing bidirectional long short‐term memory networks) automatically identifies key gait events (e.g., heel strike, toe‐off) from the joint trajectory data with high accuracy, as validated previously (Jing et al. 2023). This allows for the fully automated calculation of gait parameters without manual annotation.

### 2.3 Testing Protocol

**FIGURE 1:**
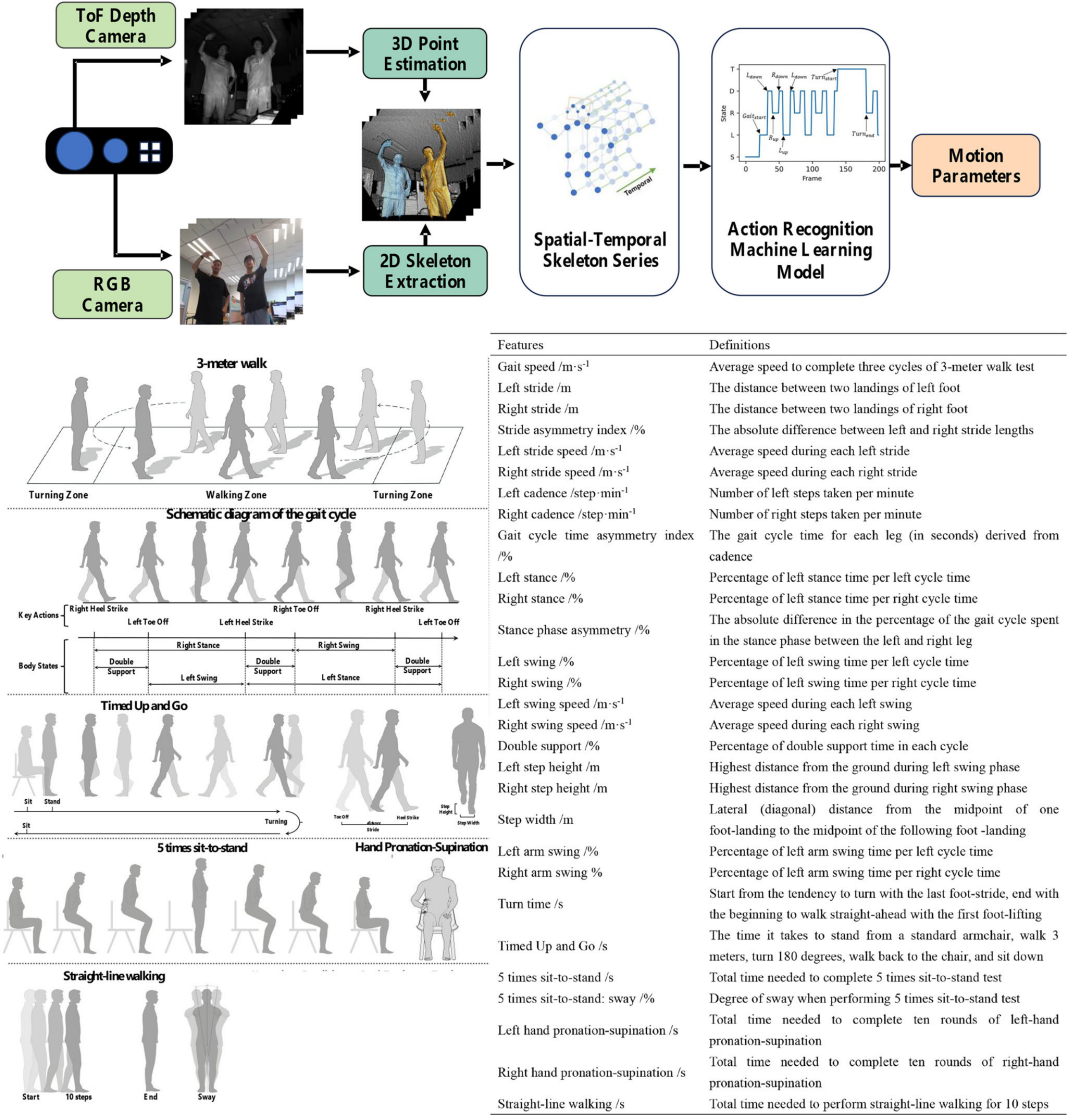
Illustration of gait parameters extraction and definitions of gait parameters.

Participants completed a standardized test battery in a well‐lit, unobstructed room (Figure 1):

1. 3 m walk test: Participants walked at their self‐selected, comfortable speed along a 3‐m walkway. The test was repeated three times, and the average value of the parameters from the middle steady‐state strides was used for analysis to eliminate acceleration and deceleration effects.
2. Timed up and go (TUG) test: Time to rise from a chair, walk 3 m, turn, walk back, and sit down was recorded.
3. Five times sit‐to‐stand (FTSTS) Test: Time to complete five consecutive sit‐to‐stand cycles was recorded.

A minimum 1 min rest period was provided between trials to prevent fatigue. All instructions were standardized.

### 2.4 Gait Parameters

A total of 20 spatiotemporal gait parameters were derived (see Supplementary Table 1 for a complete list and definitions). For analysis, parameters were grouped into five functional domains:

1. Basic spatiotemporal parameters: gait speed, stride length (left/right), stride speed (left/right), cadence (left/right), step height (left/right), and straight‐line walking time.
2. Postural stability parameters: step width, stance phase (%), double support phase (%), turn time, TUG time, stance phase asymmetry.
3. Rhythm and coordination parameters: swing phase (%), swing speed, stride asymmetry index, and gait cycle time asymmetry index.
4. Functional task parameters: five‐times sit‐to‐stand time, sway% during sit‐to‐stand, and hand pronation‐supination time (left/right).
5. Arm swing parameter: arm swing amplitude (left/right).

### 2.5 Statistical Analysis

All statistical analyses were performed using IBM SPSS Statistics (version 23.0) and JASP (version 0.19.2) software. The significance level was set at a two‐tailed *p* < 0.05.

Between‐group comparisons of demographic and clinical characteristics were performed using independent samples t‐tests or Mann‐Whitney U tests for continuous variables, and chi‐square tests for categorical variables.

To identify gait parameters independently associated with UMN dysfunction, partial correlation analyses were conducted between all gait parameters and the PENN score of lower limbs, controlling for the ALSFRS‐R total score as the primary covariate, because ALSFRS‐R is the standard measure of overall disease severity in ALS. To address potential concerns about conceptual overlap (ALSFRS‐R contains walking items), we performed sensitivity analyses controlling for total MRC score and lower limb MRC subscore. To account for multiple comparisons, we applied the Benjamini‐Hochberg false discovery rate (FDR) correction with a threshold of *q* < 0.05 to the primary analyses.

To overcome the limitation of single‐parameter analyses and to build a discriminative model, we employed a two‐step machine learning and modeling approach. First, a Random Forest classifier (500 trees) was run in JASP to rank the importance of all gait parameters and the ALSFRS‐R score in differentiating ALS‐UMN from ALS‐nUMN. The total increase in node impurity was used as the primary metric of importance. Second, the most important predictors identified by Random Forest, along with the ALSFRS‐R score (including a priori as a mandatory covariate), were entered into a least absolute shrinkage and selection operator (Lasso) regularized logistic regression model. Lasso regression was chosen for its ability to perform variable selection and handle multicollinearity by shrinking coefficients of less contributive variables to zero. We also performed 5‐fold cross‐validation to assess internal validity. The final model's predicted probabilities were used to construct a receiver operating characteristic (ROC) curve, and the area under the curve (AUC) was calculated to evaluate the model's overall discriminative performance.

## 3 Results

### 3.1 Participant Characteristics and Clinical Subgroups

A total of 118 ALS patients were finally included. Based on neurological examination, 70 patients were classified as ALS‐UMN and 48 as ALS‐nUMN. The demographic characteristics of ALS patients and HC (*n* = 1796) are provided in Table 1. Patients with UMN dysfunction tended to be younger than those without UMN dysfunction at diagnosis (55.16 ± 9.19 vs. 59.40 ± 11.00, *p* = 0.024). No significant difference in age at enrollment, gender ratio, and BMI between involved ALS patients and HC was found. Besides, no significant difference in gait parameters among age‐ and gender‐subgroups among ALS population was found as shown in Table 2 and Supplementary Table 1.

**TABLE 1:** Demographic characteristics of involved ALS patients and healthy controls.

Items	ALS	ALS‐UMN	ALS‐nUMN	ALS‐UMN vs ALS‐nUMN	HC	ALS vs. HC
*N*	118	70	48	—	1796	—
Age (years old)	56.88 (10.08)	55.16 (9.19)	59.40 (11.00)	0.024	58.98 (14.91)	0.132
Gender (M/F)	63/55	38/32	25/23	0.814	838/958	0.156
BMI (kg/m^2^)	23.48 (3.80)	23.95 (2.95)	22.80 (4.73)	0.106	24.04 (3.51)	0.096
Disease duration (months)	11 (6–17)	11 (5–18)	12 (8–17)	0.876	—	—
Bulbar onset (*n*, %)	24 (20.34%)	13 (18.57%)	11 (22.92%)	0.565	—	—
ALSFRS‐R score	41 (36–44)	41 (36–44)	41 (36–44)	0.682	—	—
Total MRC score	103 (94–112)	101 (95–109)	104 (90–111)	0.910	—	—

**Abbreviation**: ALS = amyotrophic lateral sclerosis, ALSFRS‐R ALS functional research score‐revised, BMI = body mass index, F = female, HC = healthy controls, UMN = upper motor neuron, M = male, MRC = Medical Research Council.

*Note*: Normally distributed variables were expressed as means (standard deviation, SD) and non‐normally distributed variables were expressed as medians (interquartile range, IQR). Chi square analysis or independent sample *t*‐test was used for comparisons, and significant data (*p*<0.05) were bold.

**TABLE 2:** Gait parameters in ALS patients stratified by age and compared to that in healthy controls.

Functional domain	Gait parameters	<50 years old	50‐59 years old	60‐69 years old	70‐79 years old	Inter‐group comparisons (*p*)	Overall	Healthy controls	Comparisons with healthy controls (*p*)
—	*N*	27	38	42	11	—	118	1796	—
Basic spatiotemporal	Gait speed /m·s^−1^	0.84 ± 0.30	0.87 ± 0.27	0.76 ± 0.23	0.72 ± 0.27	0.209	0.81 ± 0.26	1.25 ± 0.27	<0.001
	Left stride /m	1.00 ± 0.22	1.04 ± 0.23	0.97 ± 0.23	0.93 ± 0.22	0.487	1.00 ± 0.23	1.12 ± 0.19	<0.001
	Right stride /m	1.01 ± 0.22	1.03 ± 0.24	0.98 ± 0.23	0.94 ± 0.20	0.605	1.00 ± 0.23	1.11 ± 0.17	<0.001
	Left stride speed /m·s^−1^	0.85 ± 0.26	0.89 ± 0.29	0.79 ± 0.24	0.77 ± 0.27	0.382	0.83 ± 0.26	1.25 ± 0.27	<0.001
	Right stride speed /m·s^−1^	0.86 ± 0.27	0.88 ± 0.27	0.79 ± 0.23	0.78 ± 0.27	0.282	0.83 ± 0.26	1.25 ± 0.26	<0.001
	Left cadence /step·min^−1^	101.55 ± 12.48	100.21 ± 17.27	96.73 ± 12.80	97.70 ± 16.78	0.543	99.04 ± 14.64	138.29 ± 22.45	<0.001
	Right cadence /step·min^−1^	99.03 ± 13.17	100.36 ± 17.83	95.67 ± 13.05	96.93 ± 20.18	0.574	98.07 ± 15.41	137.08 ± 22.49	<0.001
	Left step height /m	0.11 ± 0.02	0.12 ± 0.02	0.12 ± 0.02	0.11 ± 0.02	0.735	0.11 ± 0.02	0.11 ± 0.02	0.877
	Right step height /m	0.11 ± 0.03	0.12 ± 0.03	0.11 ± 0.03	0.10 ± 0.02	0.620	0.11 ± 0.03	0.11 ± 0.02	0.579
	Straight‐line walking /s	12.13 ± 6.06	11.32 ± 3.91	11.15 ± 4.52	9.93 ± 7.50	0.667	11.31 ± 5.03	12.29 ± 3.72	0.038
Postural stability	Step width /m	0.15 ± 0.03	0.14 ± 0.02	0.14 ± 0.03	0.13 ± 0.02	0.407	0.14 ± 0.03	0.12 ± 0.02	<0.001
	Left stance /%	68.58 ± 3.05	68.42 ± 4.33	69.85 ± 3.77	68.84 ± 4.53	0.366	69.00 ± 3.90	67.06 ± 3.22	<0.001
	Right stance /%	68.66 ± 3.08	68.47 ± 4.76	69.64 ± 3.46	69.55 ± 5.17	0.553	69.03 ± 4.01	66.45 ± 3.29	<0.001
	Double support /%	38.17 ± 5.63	37.94 ± 8.13	39.30 ± 6.47	39.27 ± 9.12	0.819	38.60 ± 7.08	33.00 ± 5.29	<0.001
	Turn time /s	1.49 ± 0.48	1.43 ± 0.40	1.46 ± 0.51	1.53 ± 0.70	0.935	1.46 ± 0.49	1.11 ± 0.42	<0.001
	Timed up and go /s	15.26 ± 6.54	16.35 ± 12.00	17.09 ± 9.31	16.78 ± 6.85	0.893	16.40 ± 9.48	12.33 ± 3.09	<0.001
	Stance phase asymmetry index /%	2.48 ± 1.94	1.89 ± 1.15	2.39 ± 1.92	2.35 ± 1.99	0.471	2.25 ± 1.71	3.62 ± 3.63	<0.001
Rhythm and coordination	Left swing /%	31.41 ± 3.05	31.58 ± 4.33	30.14 ± 4.53	31.15 ± 4.53	0.366	30.99 ± 3.90	32.94 ± 3.22	<0.001
	Right swing /%	31.33 ± 3.08	31.52 ± 4.76	30.35 ± 3.46	30.44 ± 5.17	0.553	30.96 ± 4.01	33.55 ± 3.29	<0.001
	Left swing speed /m·s^−1^	2.16 ± 0.52	2.23 ± 0.44	2.04 ± 0.44	2.00 ± 0.48	0.282	2.12 ± 0.50	2.84 ± 0.48	<0.001
	Right swing speed /m·s^−1^	2.18 ± 0.53	2.22 ± 0.54	2.02 ± 0.47	1.95 ± 0.50	0.203	2.12 ± 0.52	2.83 ± 0.46	<0.001
	Stride asymmetry index /%	2.57 ± 2.01	3.16 ± 2.53	2.52 ± 1.94	3.09 ± 4.32	0.631	2.79 ± 2.43	7.33 ± 10.80	<0.001
	Gait cycle time asymmetry index /%	4.94 ± 4.07	4.33 ± 5.03	4.96 ± 4.40	6.04 ± 7.58	0.775	4.85 ± 4.86	6.24 ± 6.49	0.045
Functional task	5 times sit‐to‐stand /s	12.11 ± 5.65	11.97 ± 5.99	14.65 ± 8.81	12.60 ± 9.66	0.362	13.01 ± 7.43	12.76 ± 3.22	0.771
	5 times sit‐to‐stand: sway /%	37.38 ± 9.52	36.03 ± 11.89	37.01 ± 11.95	31.25 ± 12.24	0.468	36.24 ± 11.43	29.82 ± 6.96	<0.001
	Left hand pronation‐supination /s	8.06 ± 3.29	7.64 ± 3.37	8.53 ± 3.33	8.88 ± 3.18	0.578	8.17 ± 3.31	6.95 ± 1.93	<0.001
	Right hand pronation‐supination /s	8.41 ± 3.71	8.50 ± 3.36	8.65 ± 3.07	8.85 ± 2.67	0.980	8.57 ± 3.25	6.75 ± 1.92	<0.001
Arm swing	Left arm swing /%	36.19 ± 16.78	37.26 ± 21.02	36.91 ± 23.15	42.38 ± 36.50	0.890	37.37 ± 22.51	33.08 ± 3.12	0.031
	Right arm swing /%	37.92 ± 16.37	38.32 ± 21.70	39.68 ± 22.25	43.56 ± 34.49	0.899	39.20 ± 22.05	33.67 ± 3.22	0.009

*Note*: One‐way ANOVA analysis and independent sample *t*‐test were used for inter‐group comparisons, and significant data (*p* < 0.05) was bold.

### 3.2 Gait Abnormalities in ALS Patients vs. HC

In comparison with HC, significantly lower levels of gait speed (*p* < 0.001), step stride (*p* < 0.001), stride speed (*p* < 0.001), cadence (*p* < 0.001), swing speed (*p* < 0.001), and higher levels of step width (*p* < 0.001) and turn time (*p* < 0.001) were detected in ALS patients. During the 3 m walkway, ALS patients reported higher percentages of stance (*p* < 0.001), double support (*p* < 0.001), arm swing (*p* < 0.05) position, and lower percentages of swing (*p* < 0.001) position compared with HC. No significant difference in step height was found. The levels of the stance phase asymmetry index (*p* < 0.001), stride asymmetry index (*p* < 0.001), and gait cycle time asymmetry index (*p* = 0.045) among ALS patients were significantly lower than HC. The total time for the TUG test in ALS patients was significantly longer than that in HC (*p* < 0.001). ALS patients and HC spent comparable time completing five times sit‐to‐stand (*p* = 0.771), but the former showed a significantly higher amplitude of sway (*p* < 0.001). The time needed to complete bilateral hand pronation‐supination was also significantly longer in the ALS population than that in HC (*p* < 0.001). For straight‐line walking, ALS patients speed less time than HC (*p* = 0.038). (Table 2)

All case‐control comparisons were performed on a propensity score‐matched subset of 118 HC (1:1 matching with ALS patients). After matching, there were no significant differences in age (*p* = 0.42), sex (*p* = 0.78), or BMI (*p* = 0.35). The matched subset was used for inferential statistics; the full healthy control dataset (*n* = 1796) was only used to derive normative reference values. (Supplementary Table 5)

### 3.3 Correlation of Gait Parameters With Overall Disease Severity

As presented in Table 3, all spatiotemporal gait parameters (*p* < 0.001) and most of rhythm and coordination gait parameters (*p* < 0.05) except for the stride asymmetry index (*p* = 0.079) showed significantly positive correlations with the ALSFRS‐R score in ALS patients. Most gait parameters regarding postural stability (*p* < 0.001) except for turn time (*p* = 0.718) and the stance phase asymmetry index (*p* = 0.815) reported negative relationships with the ALSFRS‐R score. Analyses of the total MRC score showed similar results.

**TABLE 3:** Partial correlations between gait parameters and PENN score, controlling for ALSFRS‐R score.

Functional domain	Gait parameters	Partial r (controlled for ALSFRS)	*p*‐value
Basic spatiotemporal	Gait speed /m·s^−1^	−0.130	0.164
	Left stride /m	0.238	0.010
	Right stride /m	−0.217	0.019
	Left stride speed /m·s^−1^	−0.146	0.116
	Right stride speed /m·s^−1^	−0.139	0.136
	Left cadence /step·min^−1^	0.038	0.684
	Right cadence /step·min^−1^	−0.012	0.898
	Left step height /m	−0.253	0.006
	Right step height /m	−0.260	0.005
	Straight‐line walking /s	−0.060	0.519
Postural stability	Step width /m	0.234	0.011
	Left stance /%	0.088	0.345
	Right stance /%	0.162	0.081
	Double support /%	0.120	0.198
	Turn time /s	0.068	0.467
	Timed Up and Go /s	0.210	**0.025**
	Stance phase asymmetry index /%	−0.027	0.773
Rhythm and coordination	Left swing /%	−0.023	0.808
	Right swing /%	−0.162	0.081
	Left swing speed /m·s^−1^	−0.174	0.061
	Right swing speed /m·s^−1^	−0.137	0.141
	Stride asymmetry index /%	0.021	0.828
	Gait cycle time asymmetry index %	0.159	0.087
Functional task	5 times sit‐to‐stand /s	−0.146	0.117
	5 times sit‐to‐stand: sway /%	−0.047	0.618
	Left hand pronation‐supination /s	0.123	0.185
	Right hand pronation‐supination /s	0.129	0.132
Arms swing	Left arm swing /%	0.006	0.949
	Right arm swing /%	0.019	0.842

*Note*: Significant *p*‐values (<0.05) should be bold. ALSFRS‐R: amyotrophic lateral sclerosis functional research score‐revised (range: 0–48). PENN score: Penn upper motor neuron score of lower limbs (range 0–6).

### 3.4 Gait Parameters Associated With UMN Dysfunction: Partial Correlation Analysis

After controlling for the ALSFRS‐R total score (Table 3), partial correlation analysis with FDR correction (*q* < 0.05) revealed that the following gait parameters were independently associated with the PENN score of the lower limbs: decreased left/right stride length (*r* = −0.301 and −0.325, both *p* < 0.001), decreased left/right step height (*r* = −0.327 and −0.332, both *p* < 0.001), increased step width (*r* = 0.295, *p* = 0.001), increased left/right stance percentage (*r* = 0.346 and 0.219, *p* < 0.001 and *p* = 0.019), increased double support percentage (*r* = 0.283, *p* = 0.002), prolonged TUG time (*r* = 0.309, *p* < 0.001), decreased left/right swing percentage (*r* = −0.219 and −0.346, *p* = 0.019 and *p* < 0.001), and increased gait cycle time asymmetry index (*r* = 0.267, *p* = 0.004). Gait speed and stride speed showed nominal significance (*p* < 0.05) but did not survive FDR correction (*q* > 0.05). Cadence and the stride asymmetry index were not significant (*p* > 0.05).

Sensitivity analyses controlling for the lower limb MRC subscore (Supplementary Table 2) or the total MRC score (Supplementary Table 3) showed directionally consistent effects, though some parameters (e.g., double support percentage) lost significance when controlling for total MRC, likely due to over‐adjustment.

### 3.5 A Multivariate Model for Identifying ALS With UMN Signs

**FIGURE 2:**
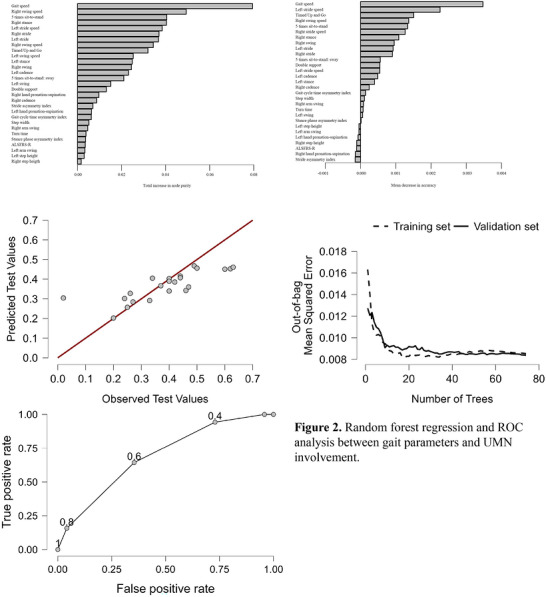
Random forest regression and ROC analysis between gait parameters and UMN involvement.

The Random Forest classifier identified the top 5–8 variables (gait speed, stride (left/right), stride speed (left), swing speed (left/right), right stance %, five times sit‐to‐stand), and the ALSFRS‐R total score as the most important features for classification. The subsequent Lasso regression model, incorporating these features, retained (left and right stride, and the ALSFRS‐R total score] as the most parsimonious predictors. This multivariate model demonstrated good discriminative ability, with an AUC of 0.690 (sensitivity 0.816, specificity 0.418) (Figure 2 and Table 4).

**TABLE 4:** Lasso regression analysis between gait parameters and UMN involvement of lower limbs.

Gait parameters	Lasso regression analysis	
	Standardized i	*p*
Gait speed /m·s^−1^	0.126	0.518
Left stride /m	−0.330	0.011
Right stride /m	−0.130	0.039
Left stride speed /m·s^−1^	0.000	0.442
Right stance /%	0.038	0.338
Left swing speed /m·s^−1^	0.000	0.472
Right swing speed /m·s^−1^	0.133	0.444
5 times sit‐to‐stand /s	−0.008	0.784

*Note*: Gait parameters with the best performance in the random forest regression were involved in the Lasso regression analysis. Significant data (*p* < 0.05) were bold.

The 5‐fold cross‐validated AUC of the Lasso model was 0.657 (SD 0.053), slightly lower than the full‑sample AUC, indicating mild optimism. At the original threshold, given a UMN prevalence of 59.3%, the positive predictive value (PPV) was 0.68, and the negative predictive value (NPV) was 0.60. (Supplementary Table 4)

## 4 Discussion

This study utilized quantitative gait analysis combined with multivariate statistical modeling to systematically investigate the neural substrates of gait disturbance in ALS. The principal findings are twofold: first, after controlling for overall disease severity (ALSFRS‐R), a specific set of gait parameters reflecting rhythm, stability, and symmetry was independently associated with the degree of lower limb UMN involvement; second, a multivariate model constructed from gait features provided an objective tool with high sensitivity for identifying a subgroup of ALS patients with clinical UMN signs.

### 4.1 General Gait Impairment in ALS

Consistent with widespread motor decline, ALS patients exhibited generalized gait deterioration compared with HC, including reduced speed, shortened stride, increased step width, and impaired dynamic balance (prolonged TUG time). This is consistent with prior studies (Iancu Ferfoglia et al. 2016; Lukac et al. 2024). Indeed, earlier work by Hausdorff et al. demonstrated that ALS patients exhibit less steady and more temporally disorganized gait rhythms compared with HC, as reflected by increased stride‐to‐stride fluctuations (Hausdorff et al. 2000). More recently, Dubbioso et al. extended these observations by showing that ALS patients with mild cognitive impairment (MCI) display exaggerated gait variabilityiability gait variabilitynce in ALS. The principal fi under dual‐task conditions, and that MCI status independently predicts short‐term fall risk (Dubbioso et al. 2023). Collectively, these studies point to a multi‐system nature of gait impairment in ALS that extends beyond pure corticospinal tract dysfunction to encompass cognitive and frontostriatal contributions.

However, a critical gap remains: the specific contribution of UMN dysfunction, independent of overall weakness and global disease severity, has not been systematically dissected in prior work. The strong correlation between gait parameters and both the ALSFRS‐R and MRC scores underscores the potential of quantitative gait analysis as an objective, continuous measure of functional status. Yet, these correlations do not disentangle whether altered gait features are driven by UMN signs (e.g., spasticity, impaired coordination) versus LMN signs (e.g., weakness, atrophy). Our study addresses this gap by applying partial correlation analyses controlling for ALSFRS‐R and MRC scores to isolate UMN‐specific gait signatures and by developing a multivariate classifier for identifying patients with pyramidal involvement.The strong correlation between gait parameters and both the ALSFRS‐R and MRC scores underscores the potential of quantitative gait analysis as an objective, continuous measure of functional status.

### 4.2 Gait Features Specifically Associated With UMN Dysfunction: Pathophysiological Insights

The core advance of this study lies in identifying a gait pattern independently linked to UMN impairment after partialling out the confounding effect of generalized weakness via partial correlation analysis. While reduced gait speed and stride length are non‐specific, they may partly stem from decreased movement efficiency due to spastic hypertonia in the context of UMN involvement. More indicative are increased step width and prolonged double support time, classic compensatory strategies for stability, likely corresponding to impaired coordination and reduced balance confidence secondary to UMN pathology. Most notably, the elevated gait cycle time asymmetry index directly reflects disrupted synchronization of left‐right locomotor rhythm, suggesting impaired integration of central pattern generators or their supra‐segmental control by the corticospinal tracts.

Our findings resonate with and extend prior observations. Hausdorff et al. first characterized stride‐to‐stride fluctuations in ALS gait (Hausdorff et al. 2000), but their analysis did not distinguish UMN from LMN contributions. More recent work by Dubbioso et al. (Dubbioso et al. 2023) identified that ALS patients with MCI show exaggerated gait variability, especially under dual‐task conditions, suggesting that cognitive‐motor interference affects locomotor control even in ambulatory patients. Importantly, the gait features we identified as UMN‐associated (increased step width, prolonged double support, elevated temporal asymmetry) share certain similarities with the “cautious gait” pattern described in patients with frontal‐subcortical dysfunction, raising the possibility that UMN pathology may co‐occur with or amplify cognitive contributions to gait instability. However, our study did not systematically assess cognition, and future studies integrating neuropsychological and neurophysiological assessments are needed to disentangle the independent and interactive effects of UMN burden, frontostriatal integrity, and cognitive status on gait in ALS.

Together, the features we identified delineate a quantifiable gait pattern associated with UMN dysfunction (characterized by widened base, prolonged double support, and increased temporal asymmetry), providing mechanistic insight into the specific role of UMN involvement in ALS gait disturbance.

We note that when controlling for the total MRC score (Supplementary Table 3), some parameters (e.g., double support percentage, gait cycle time asymmetry) were no longer statistically significant. This may reflect over‐adjustment, as the total MRC score is strongly correlated with gait parameters (*r* > 0.4), and UMN dysfunction can influence MRC testing through spasticity. The consistency of results across ALSFRS‐R and lower limb MRC adjustments supports the validity of our primary findings.

### 4.3 Clinical Implications and Exploratory Value of the Multivariate Model

The model constructed via Random Forest and Lasso regression (AUC = 0.690, cross‐validated AUC = 0.657) holds exploratory value. Its high sensitivity (0.816) suggests potential utility for screening (ruling out UMN signs when the test is negative), but the low specificity (0.418) and moderate PPV (0.68) indicate that a positive test alone is insufficient for clinical diagnosis. Therefore, we propose that this gait‐based model could serve as an ancillary screening tool or as a method for cohort enrichment in research settings, rather than a standalone diagnostic classifier. Future integration with neurophysiological or imaging biomarkers may improve specificity.

### 4.4 Limitations

This study has several limitations. First, the cross‐sectional design precludes inferences regarding causality or the prognostic value of these gait parameters for disease progression. Second, although the sample size is substantial for a single‐center gait study, all patients were recruited from a single tertiary center, and those unable to walk independently were excluded, potentially introducing selection bias and limiting generalizability to more advanced ALS stages. Third, the discriminatory performance of the multivariate model (AUC 0.690), while suggestive, is not yet excellent (AUC > 0.8), and its specificity requires improvement, necessitating validation and optimization in an independent cohort. Fourth, we lacked quantitative LMN burden measures (e.g., CMAP amplitude, motor unit number estimation) in the lower limbs. Although we controlled for the MRC score as a proxy of muscle strength, residual confounding by LMN pathology cannot be fully excluded. Therefore, a true dissociation between UMN‐pecific and LMN‐specific gait contributions requires further study with neurophysiological LMN markers. Fifth, the short (3 m) walkway, while chosen to minimize fatigue, may limit the number of steady‐state strides. The average number of strides analyzed per participant was 12.4 (SD 2.1), and intra‐test reliability for key parameters was good to excellent (ICC range 0.78–0.92). Sixth, differences in the timing and geographic source of healthy control data, despite propensity score matching, may introduce unmeasured confounding. We matched 118 controls 1:1 to the ALS cohort, and balance diagnostics showed no significant differences in age, sex, or BMI.

### 4.5 Future Directions and Clinical Translation

Despite these limitations, this study offers a novel framework for fine‐grained phenotyping in ALS. Future work should employ longitudinal designs to evaluate whether these gait parameters, particularly temporal asymmetry, can predict the progression of UMN signs or fall risk. Validation and refinement of the classification model in larger, multi‐center cohorts are essential. Clinically, this research suggests that quantitative gait analysis can serve as a valuable adjunct to traditional rating scales, particularly for objectively monitoring treatment response in patients with prominent spasticity or as an exploratory endpoint in clinical trials targeting UMN pathology.

## 5 Conclusion

In conclusion, this study demonstrates that quantitative gait analysis can identify specific locomotor features, such as increased step width, prolonged double support, and elevated temporal asymmetry, that are independently associated with upper motor neuron dysfunction in ALS beyond generic weakness. The derived multivariate model showed moderate discriminative power with high sensitivity, supporting the exploratory utility of objective gait metrics for patient screening and enriched phenotyping. However, external validation and integration with neurophysiological markers are needed before clinical application. These findings advance the characterization of ALS gait pathophysiology and encourage further development of technology‐aided motor biomarkers.

## Author Contributions

**Nan Hu**: methodology, investigation, data curation, writing – original draft, formal analysis. **Kai Wang**: data curation, investigation. **Dingding Zhang**: software, investigation. **Ming Qi**: investigation, validation. **Ning Su**: investigation, validation, visualization. **Jiangxia Zhang**: data curation. **Zhenzhong Li**: data curation, investigation. **Yuming Xu**: investigation, data curation. **Lihua Wang**: data curation, investigation. **Lan Chu**: data curation, investigation. **Yinzhou Wang**: investigation, data curation. **Yun Xu**: data curation. **Bo Hu**: investigation, data curation. **Jian Wu**: investigation. **Bo Wu**: investigation, data curation. **Hong Jiang**: investigation, data curation. **Xiangmin Fan**: investigation. **Mingsheng Liu**: conceptualization, funding acquisition, project administration, writing – review and editing. **Zhengqi Lu**: resources, investigation. **Fei Han**: investigation. **Jing Yuan**: investigation. **Feng Tian**: investigation. **Yicheng Zhu**: writing – review and editing, conceptualization, supervision.

## Funding

This work was funded by National Science and Technology Major Project (2022ZD0118003) and CAMS Innovation Fund for Medical Sciences (CIFMS 2021‐I2M‐1‐003), and was supported by the Ministry of Industry and Information Technology of China.

## Ethics Statement

The study was approved by the Ethics Committee of the Peking Union Medical College Hospital (PUMCH) (HS‐3076). All enrolled patients in our database provided written, informed consent to be included in the study.

## Conflicts of Interest

The authors declare no confluicts of interest.

## Supporting information




**Supplementary Table 1**. Gait parameters in ALS patients stratified by gender.
**Supplementary Table 2**. Partial correlations with FDR correction between gait parameters and the PENN score, controlling for the lower limb MRC subscore.
**Supplementary Table 3**. Partial correlations with FDR correction between gait parameters and the PENN score, controlling for the total MRC score.
**Supplementary Table 4**. Five‐fold cross‐validation performance of the Lasso regression model for identifying ALS patients with UMN signs.
**Supplementary Table 5**. Balance diagnostics after propensity score matching (1:1) between ALS patients and the healthy controls.

## Data Availability

The data that support the findings of this study are available on request from the corresponding author. The data are not publicly available due to privacy or ethical restrictions.
