# Supplementary material for: Quantitative Gait Analysis Reveals Distinct Patterns Associated With Pyramidal Involvement in Amyotrophic Lateral Sclerosis: A Cross‐Sectional Study
Source: Brain Behav. 2026 May 19;16(5):e71498. doi: 10.1002/brb3.71498 (PMC13184536; doi:10.1002/brb3.71498)
Supplement: Supplementary file 1 — Supplementary Table 1. Gait parameters in ALS patients stratified by gender. Supplementary Table 2. Partial correlations with FDR correction between gait parameters and the PENN score, controlling for the lower limb MRC subscore. Supplementary Table 3. Partial correlations with FDR correction between gait parameters and the PENN score, controlling for the total MRC score. Supplementary Table 4. Five‐fold cross‐validation performance of the Lasso regression model for identifying ALS patients with UMN signs. Supplementary Table 5. Balance diagnostics after propensity score matching (1:1) between ALS patients and the healthy controls. [file BRB3-16-e71498-s001.docx]

**Supplementary Table 1.** Gait parameters in ALS patients stratified by gender.

| Gait parameters | Male | Female | *p* |
| --- | --- | --- | --- |
| N | 63 | 55 |  |
| Gait speed /m·s^-1^ | 0.78±0.27 | 0.84±0.26 | 0.635 |
| Left stride /m | 1.01±0.24 | 0.98±0.21 | 0.115 |
| Right stride /m | 1.01±0.24 | 0.98±0.21 | 0.136 |
| Stride asymmetry index /% | 2.80±2.10 | 2.80±2.80 | 0.851 |
| Left stride speed /m·s^-1^ | 0.80±0.26 | 0.87±0.27 | 0.622 |
| Right stride speed /m·s^-1^ | 0.80±0.25 | 0.87±0.26 | 0.687 |
| Left cadence /step·min^-1^ | 94.72±13.49 | 104.00±14.44 | 0.568 |
| Right cadence /step·min^-1^ | 93.36±14.80 | 103.45±14.42 | 0.916 |
| Gait cycle time asymmetry index /% | 5.40±4.80 | 4.30±4.90 | 0.232 |
| Left stance /% | 69.90±4.16 | 67.98±3.59 | 0.169 |
| Right stance /% | 69.67±4.16 | 68.29±3.72 | 0.191 |
| Stance phase asymmetry index /% | 2.50±1.90 | 2.00±1.50 | 0.118 |
| Left swing /% | 30.09±3.96 | 32.01±3.59 | 0.168 |
| Right swing /% | 30.32±4.16 | 31.70±3.72 | 0.191 |
| Left swing speed /m·s^-1^ | 2.08±0.49 | 2.17±0.51 | 0.623 |
| Right swing speed /m·s^-1^ | 2.06±0.50 | 2.18±0.53 | 0.859 |
| Double support /% | 39.93±7.24 | 37.08±3.63 | 0.323 |
| Left step height /m | 0.12±0.02 | 0.11±0.02 | 0.057 |
| Right step height /m | 0.12±0.03 | 0.10±0.02 | 0.733 |
| Step width /m | 0.15±0.03 | 0.14±0.03 | 0.728 |
| Left arm swing /% | 34.43±17.64 | 40.73±26.81 | 0.318 |
| Right arm swing /% | 37.09±16.35 | 41.62±27.12 | 0.127 |
| Turn time /s | 1.47±0.45 | 1.46±0.53 | 0.497 |
| Timed Up and Go /s | 16.95±10.72 | 15.80±7.96 | 0.692 |
| 5 times sit-to-stand /s | 12.80±6.45 | 13.26±8.46 | 0.175 |
| 5 times sit-to-stand: sway /% | 37.63±11.35 | 34.64±11.40 | 0.856 |
| Left hand pronation-supination /s | 7.99±3.68 | 8.37±2.84 | 0.118 |
| Right hand pronation-supination /s | 8.64±3.62 | 8.48±2.80 | 0.063 |
| Straight-line walking /s | 11.44±5.65 | 11.17±4.26 | 0.119 |

**Note:** Independent sample t-test was used for comparisons, and significant data (*p*<0.05) were bold.

**Supplementary Table 2.** Partial correlations with FDR correction between gait parameters and PENN score, controlling for lower limb MRC subscore.

| **Functional domain** | **Gait parameters** | **Partial r**  **(controlled for lower limb MRC)** | ***p*-value** |
| --- | --- | --- | --- |
| Basic spatiotemporal | Gait speed /m·s^-1^ | -0.216 | **0.019** |
|  | Left stride /m | -0.327 | **<0.001** |
|  | Right stride /m | -0.302 | **<0.001** |
|  | Left stride speed /m·s^-1^ | -0.234 | **0.011** |
|  | Right stride speed /m·s^-1^ | -0.224 | **0.015** |
|  | Left cadence /step·min^-1^ | -0.013 | 0.888 |
|  | Right cadence /step·min^-1^ | -0.076 | 0.415 |
|  | Left step height /m | -0.292 | **0.001** |
|  | Right step height /m | -0.296 | **0.001** |
|  | Straight-line walking /s | 0.043 | 0.642 |
| Postural stability | Step width /m | 0.275 | **0.003** |
|  | Left stance /% | 0.158 | 0.089 |
|  | Right stance /% | 0.239 | **0.009** |
|  | Double support /% | 0.199 | **0.032** |
|  | Turn time /s | 0.070 | 0.456 |
|  | Timed Up and Go /s | 0.254 | **0.006** |
|  | Stance phase asymmetry index /% | 0.012 | 0.898 |
| Rhythm & coordination | Left swing /% | -0.158 | 0.090 |
|  | Right swing /% | -0.239 | **0.009** |
|  | Left swing speed /m·s^-1^ | -0.251 | **0.006** |
|  | Right swing speed /m·s^-1^ | -0.221 | **0.017** |
|  | Stride asymmetry index /% | 0.029 | 0.755 |
|  | Gait cycle time asymmetry index /% | 0.221 | **0.017** |
| Functional task | 5 times sit-to-stand /s | -0.150 | 0.107 |
|  | 5 times sit-to-stand: sway /% | -0.023 | 0.808 |
|  | Left hand pronation-supination /s | 0.125 | 0.181 |
|  | Right hand pronation-supination /s | 0.128 | 0.132 |
| Arms swing | Left arm swing /% | 0.001 | 0.989 |
|  | Right arm swing /% | 0.020 | 0.828 |

**Note:** Significant p-values (<0.05) should be bold. FDR False Discovery Rate. Lower limb Medical Research Council (MRC) subscore (range 0-40). PENN score: Penn upper motor neuron score of lower limbs (range 0-6).

**Supplementary Table 3.** Partial correlations with FDR correction between gait parameters and PENN score, controlling for total MRC score.

| **Functional domain** | **Gait parameters** | **Partial r**  **(controlled for ALSFRS)** | ***p*-value** |
| --- | --- | --- | --- |
| Basic spatiotemporal | Gait speed /m·s^-1^ | -0.130 | 0.164 |
|  | Left stride /m | -0.238 | **0.010** |
|  | Right stride /m | -0.217 | **0.019** |
|  | Left stride speed /m·s^-1^ | -0.146 | 0.116 |
|  | Right stride speed /m·s^-1^ | -0.139 | 0.136 |
|  | Left cadence /step·min^-1^ | 0.038 | 0.684 |
|  | Right cadence /step·min^-1^ | -0.012 | 0.898 |
|  | Left step height /m | -0.253 | **0.006** |
|  | Right step height /m | -0.260 | **0.005** |
|  | Straight-line walking /s | -0.060 | 0.519 |
| Postural stability | Step width /m | 0.234 | **0.011** |
|  | Left stance /% | 0.088 | 0.345 |
|  | Right stance /% | 0.162 | 0.081 |
|  | Double support /% | 0.120 | 0.198 |
|  | Turn time /s | 0.068 | 0.467 |
|  | Timed Up and Go /s | 0.210 | **0.025** |
|  | Stance phase asymmetry index /% | -0.027 | 0.773 |
| Rhythm & coordination | Left swing /% | -0.023 | 0.808 |
|  | Right swing /% | -0.162 | 0.081 |
|  | Left swing speed /m·s^-1^ | -0.174 | 0.061 |
|  | Right swing speed /m·s^-1^ | -0.137 | 0.141 |
|  | Stride asymmetry index /% | 0.021 | 0.828 |
|  | Gait cycle time asymmetry index /% | 0.159 | 0.087 |
| Functional task | 5 times sit-to-stand /s | -0.146 | 0.117 |
|  | 5 times sit-to-stand: sway /% | -0.047 | 0.618 |
|  | Left hand pronation-supination /s | 0.123 | 0.185 |
|  | Right hand pronation-supination /s | 0.129 | 0.132 |
| Arms swing | Left arm swing /% | 0.006 | 0.949 |
|  | Right arm swing /% | 0.019 | 0.842 |

**Note:** Significant p-values (<0.05) should be bold. FDR False Discovery Rate. MRC: Medical Research Council sum score (range 0-120). PENN score: Penn upper motor neuron score of lower limbs (range 0-6).

**Supplementary Table 4.** Five‑fold cross‑validation performance of the Lasso regression model for identifying ALS patients with UMN signs.

| Fold | AUC | Sensitivity | Specificity | PPV | NPV |
| --- | --- | --- | --- | --- | --- |
| 1 | 0.64 | 0.79 | 0.40 | 0.66 | 0.58 |
| 2 | 0.71 | 0.83 | 0.44 | 0.70 | 0.62 |
| 3 | 0.63 | 0.78 | 0.39 | 0.65 | 0.57 |
| 4 | 0.66 | 0.81 | 0.42 | 0.68 | 0.60 |
| 5 | 0.65 | 0.80 | 0.41 | 0.67 | 0.59 |
| Mean±SD | 0.66 ± 0.05 | 0.80 ± 0.05 | 0.41 ± 0.04 | 0.67 ± 0.03 | 0.59 ± 0.04 |

Note: Five‑fold cross‑validation was performed on the full ALS cohort (n=118) without pre‑specified train/test split.

AUC: area under the receiver operating characteristic curve; PPV: positive predictive value; NPV: negative predictive value.

The cross‑validated mean AUC (0.657) is slightly lower than the full‑sample AUC (0.690), indicating mild optimism. These results suggest that external validation is required before clinical application.

**Supplementary Table 5.** Balance diagnostics after propensity score matching (1:1) between ALS patients and healthy controls.

| Characteristic | ALS (n-118) | Matched HC (n=118) | Standardized mean difference (SMD) | *p*-value |
| --- | --- | --- | --- | --- |
| Age (years), mean ± SD | 56.88 ± 10.08 | 57.14 ± 10.25 | 0.025 | 0.42 |
| Sex (male/female) | 63/55 | 61/57 | 0.034 | 0.78 |
| BMI (kg/m²), mean ± SD | 23.48 ± 3.80 | 23.61 ± 3.62 | 0.035 | 0.35 |

Note: Propensity score matching was performed using nearest‑neighbor matching without replacement (1:1 ratio). The full healthy control dataset (n=1796) was used as the donor pool. After matching, all three variables showed no significant differences between groups (p > 0.05) and SMD values were all <0.1, indicating good balance. The matched HC subset was used for all inferential case‑control comparisons (e.g., Table 2). The full HC dataset (n=1796) was only used to derive normative reference values (mean ± SD) but not for statistical testing.
